# Metaphor and the Philosophical Implications of Embodied Mathematics

**DOI:** 10.3389/fpsyg.2020.569487

**Published:** 2020-11-02

**Authors:** Bodo Winter, Jeff Yoshimi

**Affiliations:** ^1^Department of English Language and Linguistics, University of Birmingham, Birmingham, United Kingdom; ^2^Cognitive and Information Sciences, University of California, Merced, Merced, CA, United States

**Keywords:** conceptual metaphor, embodied cognition, numerical cognition, mathematical cognition, embodied mathematics, philosophy of mathematics, SNARC, cognitive linguistics

## Abstract

Embodied approaches to cognition see abstract thought and language as grounded in interactions between mind, body, and world. A particularly important challenge for embodied approaches to cognition is mathematics, perhaps the most abstract domain of human knowledge. Conceptual metaphor theory, a branch of cognitive linguistics, describes how abstract mathematical concepts are grounded in concrete physical representations. In this paper, we consider the implications of this research for the metaphysics and epistemology of mathematics. In the case of metaphysics, we argue that embodied mathematics is neutral in the sense of being compatible with all existing accounts of what mathematical entities really are. However, embodied mathematics may be able to revive an older position known as psychologism and overcome the difficulties it faces. In the case of epistemology, we argue that the evidence collected in the embodied mathematics literature is inconclusive: It does not show that abstract mathematical thinking is constituted by metaphor; it may simply show that abstract thinking is facilitated by metaphor. Our arguments suggest that closer interaction between the philosophy and cognitive science of mathematics could yield a more precise, empirically informed account of what mathematics is and how we come to have knowledge of it.

## Introduction

Embodied approaches to cognition emphasize the role of the body, action, and sensory perception in mental processes. While there are important differences between what different researchers mean by “embodied cognition” (e.g., Wilson, 2002; Wilson and Golonka, 2013; Wilson and Foglia, 2015), all accept in some form or another that higher-level processes, such as language, are influenced by or even “structured by our constant encounter and interaction with the world *via* our bodies and brains” (Gallese and Lakoff, 2005, p. 456). Those who study embodied cognition also draw to various degrees on what are called “dynamical,” “extended,” “ecological,” “embedded,” “situated,” and “enactive” approaches to cognition (e.g., Spivey, 2007; Yoshimi, 2012; Wilson and Foglia, 2015). This set of theories is as diverse as these names suggest, but one of their common features is a shared rejection of the view that human cognition is best characterized as an abstract symbol processor.

A particularly influential approach to embodied cognition is conceptual metaphor theory (Boroditsky and Ramscar, 2002; Gibbs, 2005; Landau et al., 2010; Wilson and Foglia, 2015). According to this theory, metaphors are part and parcel of everyday thought and linguistic activity: They ground the abstract and intangible in concrete concepts accessible to the senses (Lakoff and Johnson, 1980; Gibbs, 1994; Kövecses, 2002; Lakoff, 2008, 2012). For example, linguistic and experimental evidence show that people talk and think about relatively abstract social constructs, such as intimacy, love, and personality traits, in terms of such concrete physical domains as proximity (“they are close”; “they grew apart”) or warmth (“he has a warm personality”; “that was a very cold thing to do”; IJzerman and Semin, 2010; Landau et al., 2010; Fay and Maner, 2012; Landau, 2017).

A particularly challenging test case for conceptual metaphor theory is mathematics, one of the most abstract domains of human activity, with many concepts that at first sight seem to be utterly detached from the physical realm, such as imaginary numbers and transfinite cardinals. An extensive analysis of the embodiment of such abstract mathematical structures is pursued by Lakoff and Núñez (2000) in their book *Where mathematics comes from*. Speakers commonly refer to mathematical objects, such as numbers, in metaphorical terms, for example, in referring to “high numbers,” “falling prices,” and “rising taxes” (Lakoff and Johnson, 1980, p. 15–16), or when describing arithmetical operations in terms of such concrete actions as “sliding,” “putting,” or “taking away” (Staats and Batteen, 2009).

In this paper, we consider the philosophical implications of embodied mathematics. Broadly speaking, we argue that Lakoff and Núñez were hasty in drawing some of their philosophical conclusions, but that they were right to see important connections between their work and the philosophy of mathematics, and that more careful work is needed to bring their nascent ideas to fulfillment. We consider the metaphysics and epistemology of mathematics. In the case of metaphysics, we argue that embodied mathematics is neutral in the sense of being compatible with all existing accounts of what mathematical entities really are. However, despite this, embodied mathematics may be able to motivate new approaches to the metaphysics of math that resurrect and improve on an older position known as psychologism. In the case of epistemology, we argue that the available empirical evidence collected in the embodied mathematics literature inconclusive: It does not show that abstract thinking (e.g., mathematical thinking) is grounded in or constituted by metaphor; it may simply show that abstract thinking is facilitated by metaphor. In both cases, we see that more and closer interaction between philosophy and embodied mathematics could be fruitful, and we point to gaps in the existing body of empirical research that would help to facilitate such interaction.

In more detail, the plan of the paper is as follows. In “Empirical Evidence for Embodied Mathematic” we review the large body of empirical results that supports the spatial grounding of numerical concepts. In “Where Mathematics Comes From” we highlight exactly how Lakoff and Núñez extend this work by developing an account of how more abstract domains of mathematical knowledge can be understood in terms of metaphors. For example, they claim that certain features of infinitesimal calculus are grounded in the human perception of motion and that certain features of set theory are grounded in our understanding of and interaction with bounded containers.

In “Embodied Mathematics and the Metaphysics of Mathematics” we consider how embodied accounts of mathematical cognition relate to philosophical theories concerning the ontological status of mathematical objects. We argue that accounts of mathematical *thinking* do not directly support conclusions about the metaphysical nature of mathematics, insofar as results about embodied mathematical cognition are consistent with all the main positions in the philosophy of mathematics, including Platonism and nominalism. We show that Lakoff and Núñez’s own proposal for a “mind-based mathematics” corresponds to an updated version of an existing position called “psychologism,” and we show that arguments against psychologism are not addressed by Lakoff and Núñez. However, we do believe that there is potential to address these objections and to develop their mind-based mathematics into a new position in the metaphysics of mathematics.

In “Embodied Mathematics and the Epistemology of Abstract Knowledge” we explore some gaps in how embodied mathematics treats the epistemology of abstract knowledge. This section draws on existing issues that have been debated within cognitive science and cognitive linguistics. We reframe the debate around the epistemological question of whether metaphors are *constitutive* of abstract concepts (abstract concepts could not exist without metaphor) or whether they are merely *conducive*, playing a mostly facilitative role (abstract concepts could exist without metaphor but are acquired or processed more easily with metaphors). Thus, we argue that while metaphor does play a functional role in thought processes (the conducive view), there is at present no conclusive evidence that these thought processes could not exist without metaphor (the constitutive view). Finally, based on our review of the empirical literature on embodied cognition, we urge caution when treating conceptual metaphor theory as a whole as being confirmed, when only a few small subcomponents of the theory rest on empirical evidence at this stage. This discussion is a call for empirical scientists to extend existing experimental evidence for embodied mathematics beyond basic number concepts to more abstract realms.

By pursuing work along these two lines in this paper (embodied mathematics in relation to the metaphysics and epistemology of mathematics), we hope to point to fruitful new ways of studying the relationship between mathematical thinking, the nature of mathematics, and the nature of mathematical knowledge.

## Empirical Evidence for Embodied Mathematics

We take “embodied mathematics” to be any framework which sees at least *some* aspects of mathematical thinking as being influenced by basic perceptual or sensorimotor processes. As there are hundreds of studies on the embodied grounding of mathematics, which have been extensively reviewed elsewhere (Hubbard et al., 2005; Wood et al., 2008; Fischer and Brugger, 2011; Winter et al., 2015), we will only focus on a few key findings to give the reader a flavor for this field. We specifically discuss empirical research that is most relevant to the theoretical questions discussed in later sections of this paper.

A key concept in numerical cognition research is the “mental number line,” which is supported by a well-known finding known as the “spatial numerical association of response codes (SNARC) effect.” The seminal study on this topic found that smaller numbers are responded to more quickly with the left hand, whereas relatively larger numbers are responded to more quickly with the right hand (Dehaene et al., 1993). The effect depends on one’s normal writing direction: it is reversed in those who write from right-to-left, such as Palestinians (Shaki et al., 2009) and Lebanese Arabic speakers (Zebian, 2005). The SNARC effect has been replicated more than a hundred times (Wood et al., 2008). Subsequent research suggests that the mental number line carries over to mental arithmetic in the adult mind, where addition and subtraction can be thought of as rightward or leftward movement along the number line (McCrink et al., 2007; Knops et al., 2009; Marghetis et al., 2014).

A related body of evidence has explored the vertical, rather than the horizontal axis. For example, when response buttons are aligned vertically, people respond more quickly with the top response option to sentences such as “more runs were being scored this game,” whereas they are faster in responding to “less runs …” with the bottom response (Sell and Kaschak, 2012). Similarly, reading sentences about relatively larger magnitudes facilitates subsequent detection of a visual stimulus at the top of a computer screen, whereas reading sentences about relatively smaller magnitudes facilitates subsequent detection of a visual stimulus at the bottom of a computer screen (Pecher and Boot, 2011). Woodin and Winter (2018) found that participants spontaneously orient magnitude terms such as “more” and “less” vertically, whereas they prefer horizontal arrangements for exact numerals such as “1,” “2,” “3,” and so on.

Another spatial-numerical association that is evident in both language and nonlinguistic tasks is Quantity Is Size, as evidenced by such English expressions as “tiny number,” “huger number,” “growing number,” and “shrinking number.” When asked to compare the numerical magnitudes of two numbers (say, 2 vs. 8), responses are faster if the larger number is presented in a physically larger font (Henik and Tzelgov, 1982). Similarly, judging that a group of circles contains more circles than another group is faster when the area of the circles is increased relative to the comparison group (Hurewitz et al., 2006). This mental connection between “size” and “quantity” also influences action: When people grasp for same-sized blocks with different numbers written on them, they automatically widen their grip when the written numbers are larger (Andres et al., 2008).

Both size-based and vertical ways of conceptualizing quantity are also supported by the analysis of spontaneous gestures, where English speakers tend to produce pinching gestures when talking about “tiny numbers,” and they move their hands vertically upwards or downwards to expressions such as “high number” and “low number” (Winter et al., 2013). Similarly, the gestures of mathematicians, math teachers, and students may reveal that they are thinking about math in spatial terms (McNeill, 1992, p. 164–158; Goldin-Meadow, 2005; Núñez, 2008a,b;Marghetis and Núñez, 2013), and observing gestures related to spatial conceptualizations of numbers changes subsequent mathematical thought (Marghetis, 2015; Marghetis et al., 2015).

Altogether, the current state of evidence supports the idea that when people think about numbers, horizontal, vertical, or size-based spatial representations are activated. As will be discussed below, these axial representations are directly connected to Lakoff and Núñez’s metaphor-based approach to mathematical thinking. However, the notion of “embodiment” in mathematical cognition is much wider and goes beyond considerations of metaphor. For example, it has been shown that finger gnosia (the ability to sensually distinguish between fingers and mentally represent the fingers as distinct entities) is correlated with mathematical ability in children (Noël, 2005; Costa et al., 2011; Reeve, 2011). Finger posing also primes numerical representations (Sixtus et al., 2017, 2018).

Furthermore, children’s arithmetical errors often deviate by plus or minus five from the expected outcome, which is explained as being due to internal hand-based representations, as one hand corresponds to exactly five fingers (Domahs et al., 2008). Body-based representations also explain the origin of measurement terms (Cooperrider and Gentner, 2019) and base-10 numeral systems (Ifrah, 1998; Comrie, 2013).

Embodiment in the wider sense also includes the incorporation of artifacts into mathematical thinking, such as is the case with the abacus, which leads proficient users to perform mental arithmetic using a “mental abacus” (Stigler, 1984; Frank and Barner, 2012; Barner et al., 2016). Thus, overall, there is overwhelming empirical evidence for the idea that mathematical thinking interacts with a diverse set of concrete sensorimotor representations (axial, finger-based, artifact-based, etc.).

There are, however, gaps in this body of research. The first gap is that, at present, there is mixed evidence for the functional relevance of physical representations in mathematical thinking. There is a great deal of evidence for the functional relevance of embodiment in finger counting (Noël, 2005; Costa et al., 2011; Reeve, 2011), mental abacus users generally outperform non-abacus-users in mental arithmetic (Stigler, 1984; Frank and Barner, 2012), and training children in the use of a mental abacus improves their mathematical ability (Barner et al., 2016). These results clearly show that concrete representations aid the development of mathematical concepts. However, when it comes to axial spatial representations – such as evidenced by the SNARC effect – there is relatively little evidence for any relation to mathematical ability (Cipora and Nuerk, 2013; Cipora et al., 2015, 2018). Moreover, professional mathematicians do not exhibit the otherwise widely attested SNARC effect (Cipora et al., 2016). We revisit the implications of these findings for metaphor-based views of mathematical thinking below.

Another shortcoming is that much of the existing empirical work that can be interpreted as supporting embodied mathematics has focused on relatively basic aspects of mathematics, such as our understanding of numbers and arithmetic. At its most abstract, the experimental literature has discussed the representation of negative numbers (Fischer and Rottmann, 2005; Shaki and Petrusic, 2005; Marghetis and Youngstrom, 2014). By comparison, the conceptualization of more advanced areas of mathematics (e.g., calculus, linear algebra, and number theory) is poorly understood. This was one of the major advances of Lakoff and Núñez’s book at the time: It specifically targets some of the more abstract areas of mathematics and shows how they too can be understood in terms of more concrete, grounded conceptualizations.

## Where Mathematics Comes From

Lakoff and Núñez pursue an ambitious agenda in their book: describing a system of conceptual mappings that show how a large part of mathematics might be metaphorically grounded. This is a considerable extension of the empirical work on numerical concepts discussed in the last section. Going beyond the experimental literature – which is largely focused on basic number concepts and relatively simple mathematical processes such as arithmetic – Lakoff and Núñez extend their embodied approach to concepts associated with higher mathematics, including sets, hypersets, complex numbers, limits, infinitesimals, and transfinite numbers.

### Mathematical Knowledge as a System of Conceptual Metaphors

Lakoff and Núñez’s account is firmly grounded in conceptual metaphor theory, although they also incorporate other theoretical resources associated with cognitive linguistics, including image schemas (Johnson, 1987), frames (Fillmore, 1982), blends (Fauconnier and Turner, 1998), and fictive motion (Matlock, 2004). All of these theoretical constructs are associated with an embodied understanding of language that is characteristic of cognitive linguistics (Johnson and Lakoff, 2002; Evans and Green, 2006).

A conceptual metaphor is a mental mapping between different conceptual domains (Lakoff and Johnson, 1980; Gibbs, 1994; Kövecses, 2002). The mapping is usually thought to be asymmetrical, with a more concrete, familiar, or accessible source domain mapped onto a more abstract or unfamiliar target domain (Lakoff and Johnson, 1980; Kövecses, 2002; Casasanto and Boroditsky, 2008; Winter et al., 2015).

There are many different types of metaphors that have been posited within the body of work that characterizes conceptual metaphor theory. In *Where mathematics comes from*, Lakoff and Núñez (2000, p. 53) distinguish between “grounding metaphors” and “linking metaphors.” Grounding metaphors project directly from physical experiences to fundamental mathematical concepts. An example of this is the Arithmetic Is Object Collection metaphor (Ch. 3), where arithmetical operations such as addition and subtraction are understood in terms of the manipulation of physical objects, such as adding things to a pile or taking things away from a pile. On the other hand, Lakoff and Núñez’s linking metaphors project from various abstract parts of mathematics to each other, connecting, for example, classes to predicates, numbers to points, and line segments to arcs of a unit circle. Linking metaphors are, thus, one step or several steps further removed from embodied experience than grounding metaphors. The overarching idea is that all of mathematics can be understood as a system of metaphorical mappings that are either directly grounded in experience *via* grounding metaphors or indirectly grounded *via* linking metaphors.

As is common in conceptual metaphor theory (see, e.g., Kövecses, 2002), Lakoff and Núñez allow multiple source domains to project to the same target domain. For example, arithmetic can be understood both in terms of object collection (e.g., “Take two from five and you have three left,” p. 56) and object construction (e.g., “Five is made up of two plus three” or “You can factor 28 into 7 times 4,” p. 65). Arithmetic can be understood in other ways as well. The Arithmetic Is Measuring Stick metaphor involves thinking of arithmetic as making a hypothetical measurement stick longer (addition) or shorter (subtraction). Finally, the Arithmetic Is Motion Along A Path metaphor construes numbers as points on a path that is traversed from left to right (addition) or from right to left (subtraction). Together, Lakoff and Núñez (2000, p. 75) call these metaphors the “4 grounding metaphors” or the “4 G’s” of arithmetic. Although not all of the 4G’s have been experimentally confirmed, there is, in fact, empirical evidence for the idea that people have multiple ways of conceptualizing arithmetical operations: Marghetis (2015, Ch. 4) shows that priming people with movement vs. collection-based images leads them to gesture in terms of the Arithmetic Is Motion Along A Path metaphor or the Arithmetic Is Object Collection metaphor.

**Figure 1:**
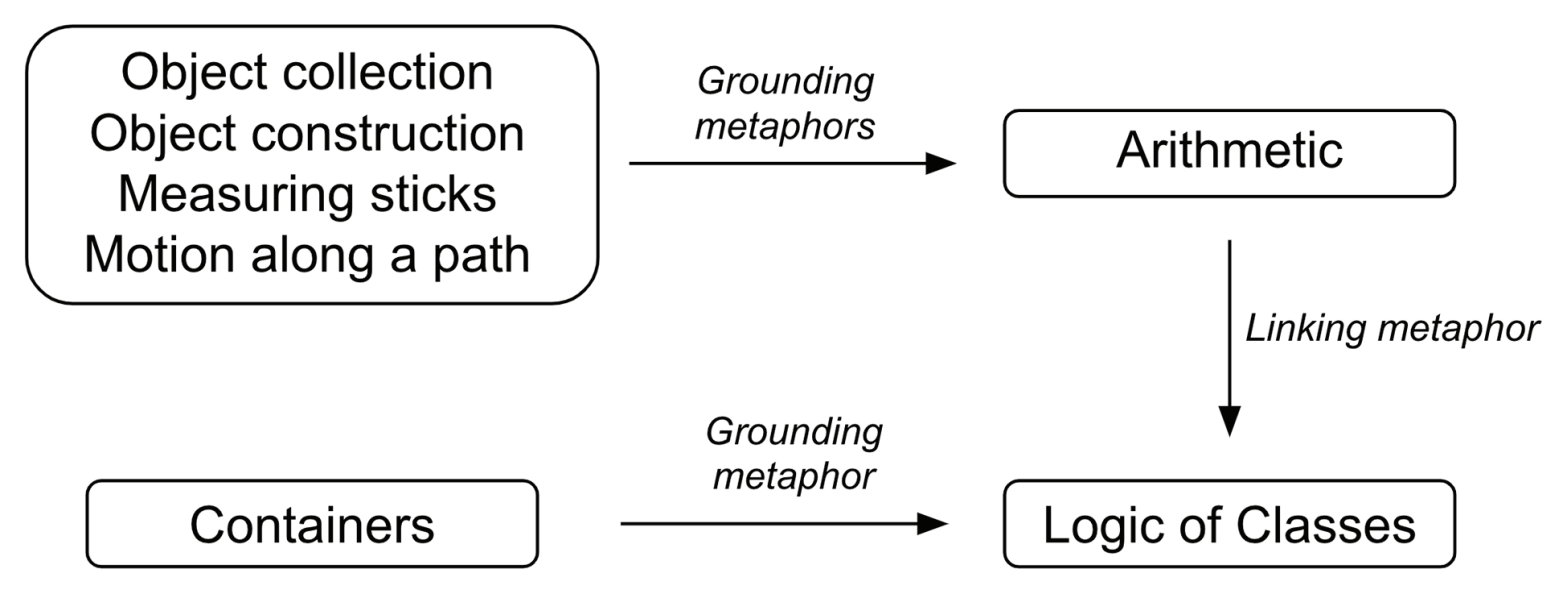
An example which shows how, according to Lakoff and Núñez (2000), different areas of mathematics are connected to each other by linking metaphors, and grounded in bodily experience.

An example of a linking metaphor is a metaphor that links basic arithmetic with Boole’s logic of classes. This metaphor maps the number “one” to a class containing a single object. It maps the operation “7 + 5” to the union of a class with seven objects and a class with five objects. As Lakoff and Núñez (2000, p. 125) describe it, this is a linking metaphor and not a grounding metaphor because “it links one branch of mathematics (the logic of classes) to another branch of mathematics (arithmetic)”. One can see how the combination of linking and grounding metaphors leads to a view of mathematics where all mathematical concepts are ultimately grounded in concrete bodily experience (see Figure 1). In this particular case, one domain of the linking metaphor, arithmetic, is itself grounded in the four grounding metaphors of arithmetic. Hence, the inferential structures that characterize the Arithmetic Is Object Collection metaphor carry over to the relatively more abstract domain of classes. On top of that, the target domain of abstract classes itself is directly grounded by the metaphor Classes Are Containers, where classes are thought of as containers and our everyday understanding of interacting with containers (putting things in them, taking things out of them) is mapped onto thinking about classes. The linking metaphor, then, links two target domains – Classes and Arithmetic – each of which is separately grounded in distinct source domains.

Within Lakoff and Núñez’s enterprise, the concept of infinity is especially important. In part, this is because infinity plays a key role in many different areas of modern mathematics, such as calculus. However, infinity is particularly important for embodied mathematics because at first sight it appears to challenge this position. How can something that is infinite be grounded by something that is bodily and, hence, finite? As Lakoff and Núñez (2000, p. 155) say, “One might think that if any concept cannot be embodied, it is the concept of infinity”.

Lakoff and Núñez dub the metaphor that grounds our understanding of infinity the Basic Metaphor of Infinity. With this metaphor, the source domain is the experience of a completed iterative process, for example, writing a series of thank you cards, or moving all of the boxes in one room to another. The target domain is an iterative process that “goes on and on,” what Aristotle called a “potential infinity” (Bowin, 2007). The crucial component of the mapping is from the part of the source domain which involves *completing* the iterative process: sending the last thank you card, or moving the last box. The final state of such a completed iterative process is mapped to the target domain of an infinitely repeating process, to yield the *metaphorical* concept of the completion of an infinitely repeating process. This is what Aristotle called an “actual infinity.” So the metaphor bridges the conceptual gap between potential and actual infinity by drawing on our fundamental experience with repeating processes that have an actual end point. The Basic Metaphor of Infinity has been further developed as a “double-scope conceptual blend” by Núñez (2005). Critical discussions of the Basic Metaphor of Infinity and several alternative accounts are reviewed in Pantsar (2015), who develops his own “process object” metaphor for infinity, whereby we map directly from our understanding of unending processes (e.g., the process of calculating successive numbers in the Fibonacci sequence) to an understanding of objects (“the” Fibonacci sequence as a mathematical object).

Lakoff and Núñez ground the Basic Metaphor of Infinity in fundamental features of the human conceptual system. Ongoing vs. completed processes are related to what linguists call *aspect* (Comrie, 1976; Matlock, 2011). For example, the sentence “He took hush money” implies a unitary event. By contrast “He was taking hush money” has a different temporal structure: It implies a repeated or iterative process (Fausey and Matlock, 2011). The interplay between potential and actual infinity is related to a more general human tendency to think of processes in terms of things, and things in terms of processes, as evidenced, for example, by the phenomenon of fictive motion, where a static location, such as a road, is understood in a dynamic fashion, such as when saying “The road runs along the coast” (Matlock, 2004). The fact that humans can easily shift between conceptualizing events as repeated and ongoing or as static and completed further supports the Basic Metaphor of Infinity as one where an endlessly repeating process is understood as having a static end point. Besides resting on the cognitive capacity to readily switch between different aspectual conceptualizations of events, the Basic Metaphor of Infinity rests on the capacity to perform mappings between domains, e.g., when repeated processes with an end are mapped onto repeated processes without an end.

### Lakoff and Núñez on the Philosophy of Mathematics

The focus of Lakoff and Núñez’s work is on mathematical cognition. Both their work and its subsequent development (Núñez, 2005; Pantsar, 2015, 2018) provide broad support for the idea that mathematical thinking is in many ways embodied. However, Lakoff and Núñez also develop an account of the ontology or metaphysics of mathematics. This metaphysical strain runs through the entire book. In the preface they say they will sketch a “beautiful picture… of what mathematics really is” (p. 18). They end by describing their results as “a theory of the only mathematics we know or can know … a theory of what mathematics is—what it really is!” (p. 346). They frame many of their analyses of mathematical ideas as also being results about mathematics itself. For example, they conclude their discussion of algebraic metaphor systems by proclaiming: “what we learn from this is not any new algebra but, rather, what algebra is” (p. 119, their emphasis).

According to Lakoff and Núñez, mathematical objects correspond to the kind of embodied conceptual structures reviewed above:

Mathematical objects are embodied concepts – that is, ideas that are ultimately grounded in human experience and put together *via* normal human conceptual mechanisms, such as image schemas, conceptual metaphors, and conceptual blends (p. 386).

Thus, for example, they say: “numbers are in people’s minds, not out in space” (p. 345). In their discussion of Cantor’s diagonalization proof, they say that the proof is “inherently metaphorical” and based “not in the external, objective world but only in minds” (p. 212).

Lakoff and Núñez contrast this view with what they call the “Romance of Mathematics,” a list of positions they take to be typically held by mathematicians and philosophers of mathematics. This “Romance of Mathematics” view can broadly be characterized as the position that mathematics has a mind-independent status, i.e., mathematics objectively exists, even without human involvement. In contrast to this view, Lakoff and Núñez (2000, p. 15) deny that mathematics has “an objective existence … independent of and transcending the existence of human beings.”

It is tempting to assume that this position slips into relativism, the idea that mathematics can be different for different cultures or different people. However, Lakoff and Núñez (2000, e.g., p. 9 and p. 362–363) also distance themselves from what they dub “radical social constructivism,” or more generally postmodern interpretations of mathematics, which see mathematics as a purely subjective cognitive enterprise that is culturally relative. Against such radically constructivist accounts, they note that the core body of mathematics appears to be relatively stable across histories and cultures, something that they argue has to do with the fact that many of our most fundamental embodied experiences (such as interactions with object collections) are shared across cultures and have not changed significantly over time. It is clear that there is important cultural variation in mathematical thinking (e.g., Stigler, 1984; Göbel et al., 2011; Bender and Beller, 2012), however, most of this variation concerns differences in low-level representation of numbers or arithmetical operations which, although they may differ in terms of processing cost and efficiency, yield mathematically equivalent results. In contrast, professional mathematicians form an international community that uses a widely shared system of mathematics.

## Embodied Mathematics and the Metaphysics of Mathematics

We have seen that Lakoff and Núñez (2000, p. 8) present their work as an account of what “mathematics really is.” Mathematical objects are taken to be embodied concepts in the mind. They present this view of *mind-based mathematics* as a new contribution to the landscape of traditional philosophy of mathematics, which “is not consistent with any of the existing philosophies of mathematics.”

In this section, we critically assess the claim that embodied mathematics provides a new answer to the question of what mathematics is. We begin by reviewing the main positions in the metaphysics of mathematics (“Platonism and Nominalism”), organizing our review around two dominant positions: Platonism and nominalism. In “The Metaphysical Neutrality of Embodied Mathematics Research” we argue that the cognitive science of mathematics is metaphysically neutral, in the sense of being compatible with all existing positions regarding the ontological status of mathematical entities. All of the results about embodied mathematics surveyed above could be endorsed by Platonists and nominalists in all of their varieties. In “Psychologism” we consider the ontological position that comes closest to Lakoff and Núñez’s mind-based view: psychologism. The problems that psychologism faces are formidable and show just how hard it is to pursue ontology using any type of mind-based mathematics, including one that is based on embodied minds. However, in “Reviving Psychologism?” we consider one way an embodied, neo-psychologist approach to mathematics might be developed by treating mathematics as the operation of universal cognitive structures that evolved to accurately represent physical regularities in the universe. Such a position still faces challenges, but the challenges could be surmounted, resulting in a plausible form of mind-based mathematics in the spirit of Lakoff and Núñez’s original work.

### Platonism and Nominalism

Within the broad landscape of philosophy of mathematics, Lakoff and Nuñez focus on the *ontology* or *metaphysics* of mathematics. What kinds of things are numbers or differential equations or sets? What is the ontological status of the objects referred to in mathematical and logical discourse? We will not canvas all extant position in the metaphysics of mathematics but will instead focus largely (but not exclusively) on the two most prominent positions: Platonism and nominalism.^1^

Platonism is the view that mathematical objects, such as numbers and sets, exist as abstract objects or *abstracta*, that is, as non-spatio-temporal objects. In the cartoon version of this view, mathematical objects exist in a “Platonic heaven” of abstract entities, a so-called “third realm” distinct from the subjective and physical realms. In more contemporary “structuralist” versions of Platonism, mathematical objects are taken to be structures or patterns instantiated by physical objects [structuralism in this sense is sometimes called *ante rem* structuralism and is contrasted with others forms of structuralism, some of which are nominalist (Shapiro, 1996)].

A standard way to support Platonism is *via* an “indispensability argument” (Putnam, 1975; Quine, 1980). Here is a standard reconstruction due to Colyvan (2012):

(P1) We ought to have ontological commitment to all and only the entities that are indispensable to our current best scientific theories.
(P2) Mathematical entities are indispensable to our best scientific theories.
(C) We ought to have ontological commitment to mathematical entities.

There are several classic problems with Platonism (Balaguer, 2009; Colyvan, 2012, section 3.3; Linnebo, 2018, “Embodied Mathematics and the Metaphysics of Mathematics” section). Perhaps the most prominent is that positing the existence of abstract, non-spatio-temporal entities conflicts with our standard physicalist picture of the world. A more specific form of this objection is epistemological (Benacerraf, 1973). According to a standard view of knowledge, when an agent has knowledge of an object, this is in part because the object causes them to have that knowledge by stimulating their sensory apparatus. But abstract entities cannot be causally connected to our sensory apparatus.

Platonism is the most prevalent form of realism about mathematical objects: it holds that mathematical objects exist as non-spatio-temporal, abstract objects. Other kinds of realists hold that mathematical objects exist as mental entities (psychologism) or physical entities (physicalism).^2^ These other forms of realism face serious problems and in light of this are minority positions in the current philosophical landscape. However, as we will see, Lakoff and Núñez may show how embodied mathematics could be used to successfully revive a position that merges psychologism with certain elements of physicalism.

Nominalism is the view that mathematical objects exist “in name only.” We have words for mathematical objects, but those words do not imply the existence of mathematical objects that exist outside of space and time. Nominalism is a popular position because it is consistent with the standard ontology of contemporary science, according to which the world is populated by physical entities, and nothing else. Nominalism is a form of *anti*-realism, which denies that mathematical statements refer to real things. Anti-realism and nominalism come in many forms (Bueno, 2013). For example, game formalism is the view that mathematics is a system of rules which involves deriving consequences from axioms in a formal system. Setting up a system of axioms is like setting up the rules of a game. There are “moves” that are consistent with those rules and moves that are not. Describing such a system of legal and illegal moves does not commit us to the existence of abstract mathematical objects. The mathematics of group theory does not commit us to the existence of abstract groups any more than chess commits us the existence of abstract chess positions.

Another, more popular form of nominalism in the contemporary literature is fictionalism (Balaguer, 2009), which sees existential mathematical statements as important falsehoods. The advantage of fictionalism is that it accepts both premises of the indispensability argument, which suggest that our best scientific theories imply the existence of mathematical objects, but then denies that those objects exist, so that our best theories are false. Of course, the idea that mathematical statements are false is jarring at first, but its adherents downplay the concern. Mathematical discourse *would* be true if abstract objects existed, and that “virtual truth” is enough to preserve all the valuable functions mathematics serves: “it’s this virtual truth, or for-all-practical-purposes truth, that’s really important. Literal truth, on this view, just is not very important; it is not to be valued; and so it just does not matter if our mathematical and scientific theories aren’t literally true” (Balaguer, 2014, p. 8).

Further discussion of game formalism, fictionalism, and other forms of nominalism are developed in (Colyvan and Zalta, 1999; Balaguer, 2009, 2018; Bueno, 2013; Weir, 2020).

### The Metaphysical Neutrality of Embodied Mathematics Research

Embodied mathematics, including the entire body of results discussed in Lakoff and Nuñez’s *Where mathematics comes from*, as well as its elaboration in the more recent literature, is consistent with Platonism, nominalism, and indeed every account of the metaphysics of mathematics that we are aware of. It is in this sense metaphysically neutral. This neutrality with respect to metaphysics does not entail that embodied mathematics is neutral with respect to other areas of inquiry in the philosophy of mathematics, such as epistemology.

One generic way to see this is to note that many contemporary philosophers of mathematics are naturalists who accept that the natural sciences – including the cognitive sciences – are our best sources of knowledge about the physical universe and the laws which govern it. Naturalist considerations are a main factor guiding nominalism, which denies the existence of abstract objects altogether. Contemporary Platonism is largely motivated by the indispensability argument, which bases the existence of mathematical objects on the fact that they are referred to by our best scientific theories. Insofar as they are naturalists, contemporary nominalists and Platonists would both accept the claims about mathematical thinking surveyed in “Empirical Evidence for Embodied Mathematic” insofar as they are supported by empirical evidence.

To further see how Platonism and nominalism are consistent with embodied mathematics, consider how empirical results in this field of study would actually look to proponents of these views. For example, consider the evidence that our fingers make it relatively easy to grasp the abstract truths of basic arithmetic, while transfinite cardinals are more difficult to grasp as they have no immediate correspondence with the physical world. Thus, we are prone to make errors like assuming that infinity is less than infinity plus one (Pantsar, 2015). So embodied mathematics shows us when it is easier or harder to grasp abstract objects, and which cognitive structures and processes facilitate or hinder the development and comprehension of new mathematical concepts. A Platonist could accept all of these results as empirically established facts about when it is easier or harder to “grasp” abstract mathematical entities, e.g., (for an *ante rem* structuralist) the structural patterns instantiated by physical objects. In a similar way, a game formalist would accept that our fingers make it easier to learn the rules of the game of basic arithmetic and that it is correspondingly harder to learn the rules of the game of transfinite arithmetic. Similar considerations show that other forms of Platonism and nominalism could also accept the results of embodied mathematics. In each case, the question of how we come to understand mathematical entities is orthogonal to the question of what those entities are or indeed whether they exist or not.

Thus, Lakoff and Núñez (2000, p. 9) are wrong to claim that their account “is not consistent with any of the existing philosophies of mathematics.” No philosophers of mathematics, on the basis of their metaphysical theories, have reason to deny that mathematical cognition is influenced by human embodiment.

### Psychologism

Psychologism is the view that the existential statements of logic and mathematics refer to psychological or cognitive processes. Psychologism emerged in England and Germany in the 19th century and is probably best known as a position Frege and Husserl argued vigorously *against* (Frege, 1884; Husserl, 2012), and by the early 20th century, it was effectively defeated as a philosophical position. It is now often remembered as a kind of mistake. As Kusch notes in a recent review: “many authors use the term ‘psychologism’ for what they perceive as the mistake of identifying non-psychological with psychological entities” (Kusch, 2020).

The simplest and least plausible version of psychologism holds that mathematical objects correspond to *actual* thought processes, which can vary from individual to individual or for one individual over time. This version of psychologism immediately raises the problem of relativism. It seems to imply, for example, that “there would be different numbers two for each person” (Frege, 1884, p. 37; Pelletier et al., 2008, p. 25). Husserl (2012, p. 78), in his critique of psychologism, devotes little attention to this form of the view, describing it as a kind of “subjectivism” that is so problematic “it is doubtful whether anyone seriously holds it.”

A more plausible form of psychologism takes mathematics to be about the thought processes of an “ideal cognizer” (Pelletier et al., 2008) and the ideal cognizer’s *possible* mental activities. This could be called a “possibilist” form of psychologism as contrasted with an actualist form of psychologism (Balaguer, 2009). Possibilist psychologism preserves the idea that mathematics is about something – namely, the abstract thought processes of an idealized agent – while avoiding the problem of relativism by claiming that mathematics is about a realm of possible thoughts which exist independently of any particular person, place, or time. However, the view seems to reduce to Platonism: the possible thoughts of an ideal cognizer are *non*-spatio-temporal abstract entities. Hence, the view must contend with the objections to Platonism noted above.

Another option is to pursue a form of psychologism according to which logic and mathematics do not correspond to possible *states* of minds but rather to something more general, e.g., a particular type of cognitive architecture. On this type of view, the truths of mathematics are based on the proper functioning of a human “logic and mathematics module” (Pelletier et al., 2008, p. 50), or (in light of problems with the concept of an isolated cognitive module) some more generic type of mental processing, i.e., a non-modular cognitive architecture (Spivey, 2007). Another approach along these lines is to draw on Chomsky’s distinction between the competence of an idealized reasoner (as contrasted with her actual performances), and then to treat mathematical competence as “the result of the socio-culturally structured acquisition of mathematical cognitive practices that capitalize on the bodily manipulation of symbolic and diagrammatic structures” (Fabry and Pantsar, 2019, p. 3).

However, it is not clear whether these refinements of psychologism are sufficient to avoid the problems that defeated it in the early 20th century. A view which focuses on cognitive architecture still locates mathematics in specific mental structures. Even if all humans share this architecture, we can imagine other intelligent creatures with different mental architectures that might support different mathematics. As Husserl noted, views like this imply that “the same proposition … can be true for a subject of the species *homo*, but may be false for another subject of a differently constituted species” (Husserl, 2012, section 36). This is counter-intuitive: The statement “2 + 2 = 4” seems to be true everywhere and at all times, regardless of a creature’s constitution.

Psychologism faces other problems as well. For example, it “seems incapable of accounting for any talk about the class of all real numbers, since human beings could never construct them all” and “seems to entail that assertions about very large numbers (in particular, numbers that no one has ever thought about) are all untrue” (Balaguer, 2009, p. 82). These may or may not be serious problems but they are standard in the literature and, thus, any form of mind-based mathematics must address them. One issue with Lakoff and Nuñez’s *Where mathematics comes from* then, is that long-standing arguments such as these are not dealt with, even though their position is most closely aligned with psychologism.

### Reviving Psychologism?

Lakoff and Nuñez seem to be in a bind. They want to emphasize a form of mind-based mathematics, according to which mathematics arises directly from its embodiment in metaphors, image schemas, and related structures in the human conceptual system, with mathematical objects having no existence independent of human cognizers. But they also want to avoid the kinds of relativistic consequences associated with social constructivist views. However, they do not show how these conflicting goals can both be satisfied. In particular, it is not clear how they can avoid relativism if they locate mathematics in the embodiment of minds like ours, since mind-based mathematics seems to lead to “species-specific relativism” or “species relativism” (Husserl, 2012; Kusch, 2020), according to which the claims of mathematics are only true for creatures embodied like we are.

However, Lakoff and Núñez’s work does suggest a way of improving on more traditional psychologist positions. They argue that mathematics works so well at describing the physical world because mathematicians have repeatedly sought to “fit” mathematics to the real world: “human physicists are successful in fitting human mathematics as they conceptualize it to their human conceptualization of the regularities they observe in the physical world” (p. 345–346). This can be thought of as a process of cultural evolution that narrows the gap between mind-dependent mathematics and mind-independent physical regularities. This approach avoids the relativistic worries that plague psychologism by anchoring mathematical cognition in objective features of the world. Different beings with different bodies should evolve similar mathematics, given that they use mathematics to describe features of the same physical world. The position is thus a form of realism that encompasses not just embodied cognitive processes but also the “regularities in the universe independent of us” (p. 355) that those thought processes are adapted to.

This view is in seeming tension with Lakoff and Núñez’s stated aversion to the idea that “the mathematics of physics resides in physical phenomena” (p. 340), though it may be acceptable to them insofar as mathematics is located not in the physical phenomena themselves but in embodied cognitive architectures which evolved to represent those objective regularities in the physical world. This view still faces further challenges (e.g., problems about very large numbers), but it does shows that, even if Lakoff and Núñez are wrong to claim that their view contradicts existing accounts of the ontology of mathematics, they may still have the resources to produce a viable new mind-based approach to mathematical ontology, an embodied form of psychologism. The results of embodied mathematics do not conclusively support this account (in light of the argument of “The Metaphysical Neutrality of Embodied Mathematics Research” section), but, nonetheless, its close alignment with the epistemology of mathematical knowledge may provide some reason to prefer it over alternative accounts.

## Embodied Mathematics and the Epistemology of Abstract Knowledge

In this section, we turn from metaphysics to epistemology, where embodied mathematics is more directly relevant to the philosophical issues (e.g., Pantsar, 2015). The question of how we come to have knowledge of mathematical truths is intimately tied to the question of how we in fact develop and use abstract knowledge, and some have explicitly argued for a “cognitively informed epistemology of mathematics” (Pantsar, 2018, p. 292). The question of the degree to which mathematical thinking requires metaphor has been independently raised in cognitive linguistics and conceptual metaphor theory. In this section, we critically revisit these questions, urging caution in the philosophical interpretation of existing empirical evidence.

### Are Metaphorical Target Domains Constituted by Source Domains?

The idea that mathematical knowledge cannot exist on its own is fundamental to Lakoff and Nuñez’s version of an embodied philosophy of mathematics. All mathematical knowledge is linked to source domains either directly *via* grounding metaphors or indirectly *via* linking and grounding metaphors. Thus, according to them, we could not have sets or differential equations without the relevant source domains. On this view, metaphors are *constitutive* of mathematical concepts. This taps into an issue that has been widely discussed within the literature on cognitive linguistics, which is whether target domains are constituted by source domain information. In this section, we draw a distinction between the view that metaphors are *constitutive* of their target domains (i.e., thinking about the abstract target domain cannot exist without metaphor) and the view that metaphors are *conducive* for target domain thinking (i.e., metaphors change or help aspects of the way target domains are conceptualized, but the target domain is cognitively realized at least partially without metaphorical mappings). Often, characterizations of metaphor theory use language that is not precise enough to establish whether a constitutive or conducive view of metaphor is held. For example, Gibbs and Berg (1999, p. 618) note that “Several kinds of evidence from psycholinguistics supports the idea that embodied metaphors underlie people’s understanding of abstract concepts.” While there is indeed evidence for the idea that metaphor facilitate people’s understanding of abstract concepts (as reviewed in “Empirical Evidence for Embodied Mathematic” section), it is not clear that metaphors “exhaust” abstract concepts. The notion that metaphors “underlie” concepts could mean that they provide helpful means of accessing those concepts, or it could mean that those concepts require the metaphor in order to exist in the first place. This is an important distinction that is masked by saying that metaphors “underlie” or “ground” abstract concepts.

Several authors have questioned the idea that metaphors are, in fact, constitutive of target domain knowledge. Barsalou (1999, p. 600) discussed the metaphor Anger Is Heated Fluid In A Container (evidenced by such English expressions as “he blew his stacks” and “I am boiling with anger”), about which he said:

Knowing only that anger is like liquid exploding from a container hardly constitutes an adequate concept. If this is all that people know, they are far from having an adequate understanding of anger.

On top of that, Barsalou (1999) noted that the idea of a “mapping,” inherent to conceptual metaphor theory, appears to logically necessitate the existence of two domains. Specifically, he noted that “a concrete domain cannot be mapped systematically into an abstract domain that has no content” (p. 600).

There is clearly evidence showing that thinking about abstract domains automatically engages concrete source domain knowledge in a way that is predicted by conceptual metaphor theory (Gibbs, 1994; Boroditsky and Ramscar, 2002; Gibbs et al., 2004; Casasanto and Boroditsky, 2008). However, the fact that metaphors become activated in particular thought processes do not say that metaphors are *necessary* for such thought (see also Murphy, 1996, 1997).

In a similar fashion to Barsalou (1999), Haser (2005, p. 157) discusses the metaphor Argument Is War. She points out that the idea, central to an embodied view of metaphor, that metaphors are acquired through environmental correlations, such as noticing similarities between arguments and fights, “presupposes an antecedent conception of what arguments are otherwise the purported ability to experience ‘correlations’ between the two domains appears wholly mysterious.” In a response to this concern, Kertész and Rákosi (2009) note that most of the writing in Lakoff and Johnson’s (1980) original work actually argues for partiality of mapping, i.e., a metaphor may concretize or embellish a particular target domain but does not fully exhaust the target domain. It seems that this partiality was largely given up for *Where mathematics comes from*, as well as for the related philosophical work presented in *Philosophy in the flesh* (Lakoff and Johnson, 1999). Thus, similar to Haser (2005), Kertész and Rákosi (2009) suggest that target domain knowledge does exist independently of metaphor, but it is incomplete with metaphor filling in gaps by mapping structures from a richer source domain. This is precisely what we mean when we say that metaphors are *conducive* rather than *constitutive* of target domain thinking.

It is also important to stress that the theoretical move by Lakoff and Nuñez to disallow the target domain a cognitive status on its own is at odds with how metaphor is usually treated in empirical research. Take for example, Thibodeau and Boroditsky’s (2011) seminal experiment which showed that describing crime with the metaphorical frame of a Beast leads participants to make different policy recommendations than if crime is described as a Virus. The idea that two different source domains highlight some elements of the target domain at the expense of others (“framing”) is based on the premise that people have some knowledge about the target domain to begin with. Similarly, we would not want to say that because there are metaphors such as Love Is A Journey, people have no other way of conceptualizing love without metaphor. Yet, this is precisely the reasoning step that Lakoff and Núñez undertake in *Where mathematics comes from* when they argue that mathematical objects have no existence without metaphor.

Future research would need to show to what extent thinking about abstract concepts is in fact, *constituted* by metaphor. The available empirical evidence clearly shows that metaphor changes reasoning and may also aid acquisition (Casasanto and de Bruin, 2019), but none of this directly supports the conclusion that reasoning and concept acquisition could not happen without these metaphors, even if reasoning without metaphor would be impoverished. More problematically for embodied mathematics is the fact that the available empirical evidence has failed to find a concrete link between mathematical achievement and thinking about numbers spatially (Cipora and Nuerk, 2013; Cipora et al., 2015, 2016, 2018). In fact, some studies have even found that stronger spatial-numerical associations are associated with *worse* mathematical ability (Kramer et al., 2018). At least on the surface, this evidence is at odds with the idea that the mental number line (which is part of Lakoff and Nuñez’s 4Gs) plays a formative role in mathematical thought. Alternatively, the results by Kramer et al. (2018) could be interpreted as being consistent with a role of the number line in scaffolding mathematical concepts, which is left behind later, for example, at the expense of more abstract symbolic representations.

It is also important to point out that for many researchers in the general cognitive science literature, metaphor is not considered to be the primary cognitive support of abstract representations. How people come to have abstract concepts is one of the key challenges of cognitive science (Kousta et al., 2011; Vigliocco et al., 2013; Borghi et al., 2017; Bolognesi and Steen, 2018; Lupyan and Winter, 2018). Most researchers in this field acknowledge that there are multiple aspects of our cognition that support abstract concepts, and a large number of people think that a lot of abstract knowledge is represented in a linguistic format (Lupyan and Winter, 2018). This is not to say that metaphor is not important for abstract thought, but the empirical literature clearly shows that it is not the only factor to consider.

To clarify: we do not mean to say that metaphors are not important for abstract thought – the available empirical evidence (see “Empirical Evidence for Embodied Mathematic”) clearly suggests that they are. We thus do not question the idea that metaphors are functionally relevant for some aspects of our mental lives, such as making it easier to think about particular topics or facilitating the acquisition of particular concepts; however, such a view is a far shot from saying that concepts *cannot* be thought without metaphor, which is indeed a claim that is hard to address with empirical evidence. Lakoff and Núñez’s argumentative move to disallow mathematics any existence of its own rests on the idea that certain aspects of abstract thought cannot exist without metaphors, and it is precisely this aspect of conceptual metaphor theory that has little empirical support at this stage.

Finally, it should be emphasized again that while there is abundant evidence for embodied effects in mathematical thinking, much of the existing literature is focused on relatively low-level number concepts and at most, simple arithmetical computation (McCrink et al., 2007; Knops et al., 2009; Marghetis et al., 2014). Some insightful work has investigated more abstract concepts, such as limits and infinity, using gesture analysis (Marghetis and Núñez, 2013), and there also is evidence for embodied representations of more abstract concepts coming from the analysis of classroom discourse (Staats and Batteen, 2009). However, these strands of evidence for the embodiment of more abstract mathematical processes are observational, with more experimental work needed that goes beyond low-level mathematics to do justice to Lakoff and Núñez’s key contribution. To make this possible, more experimental research needs to study the cognition of professional mathematicians, as has been done in only very few studies so far (Cipora et al., 2016).

### Lack of Empirical Evidence for More Complex Aspects of Metaphor Theory

In response to Rakova’s (2002) critique of their philosophical work, Johnson and Lakoff (2002) point out that their position rests on a firm body of empirical evidence supporting the idea that the mind is embodied. This argument extends to Lakoff and Núñez’s *Where mathematics comes from*. However, just because *some* aspects of metaphor theory have been empirically confirmed, we should not think that *all* of metaphor theory is backed up by empirical evidence.

For example, Haser (2005) discusses the idea that complex metaphors, such as Theories Are Buildings, are built from primary metaphors (Grady, 1997), a key component of conceptual metaphor theory that has conceptual parallels in Lakoff and Núñez’s work how grounding metaphors (such as the 4G’s) allow building up a larger cognitive edifice of mathematical thought through the extension of linking metaphors. Although theoretically appealing, none of these ideas have been tested directly, as discussed by Haser (2005). Similarly, the idea that our understanding of infinity rests on some form of metaphorical mapping is theoretically appealing and has fruitfully been applied within philosophy of mathematics (Pantsar, 2015). However, there are, at present, no direct empirical investigations of this topic that we are aware of. This is not a fault of Lakoff and Núñez’s work and its extensions, which is intended to be theoretical. It is equally an issue with the experimental research, which tends to focus on those issues that allow formulating simple experiments, which is often easier to do for simple numerical representations and low-level arithmetic. Devising experiments that test the idea that our understanding of infinity rests on aspectual thinking, or that our understanding of sets rests on containers, is a much harder task. From this perspective, Lakoff and Núñez’s book can be seen as a call for experimental researchers to tackle more abstract aspects of mathematical thinking. The book is rife with hypotheses and conjectures to be tested experimentally that would help move the field of embodied mathematical cognition research away from exclusively focusing on basic number concepts.

The arguments developed above put pressure on the claim that metaphor is constitutive for abstract thinking, including mathematical thinking, which is another aspect of metaphor theory that is in further need of empirical investigation. However, conversely, our discussion leaves open the possibility that there are metaphors that are not only conducive of abstract thought but also constitutive of it. Further empirical work testing these questions, in coordination with philosophical work in the relevant areas of epistemology, is needed to make progress on these issues.

## Conclusion

Embodied approaches to numerical and mathematical thought have produced an impressive array of results, which promise to yield new insights into the nature of mathematical knowledge and even mathematics itself. However, in both cases, we urge caution. The empirical results are strictly speaking neutral with respect to the ontological status of mathematical objects, and the available empirical evidence may only show that metaphor is conductive to abstract thought, rather than constitutive of it. In addition, we have warned against treating conceptual metaphor theory as a whole as empirically confirmed when only some parts of it rest on empirical evidence. However, all of this points to exciting new directions for research in the cognitive science and philosophy of mathematics. Existing work on the embodied representation of number could be extended to more abstract domains of mathematics. Studies could be designed to test the difference between the “constitutive” and “conducive” views of metaphor. The revival of psychologism outlined above could be further developed in order to see how it fares with respect to the full battery of counter-arguments that might be made against it. In these and other ways, our hope is to motivate a closer integration between accounts of how mathematical thinking actually unfolds, what mathematical objects are, how we come to have knowledge of them, and how to experimentally probe this knowledge.

## Author Contributions

BW and JY co-developed the argument and co-wrote the manuscript. Both the authors contributed to the article and approved the submitted version.

### Conflict of Interest

The authors declare that the research was conducted in the absence of any commercial or financial relationships that could be construed as a potential conflict of interest.

## Footnotes

^1^Contemporary philosophers are evenly divided between these positions (Bourget and Chalmers, 2014). In a 2009 survey of 931 professional philosophers, 39.3% said they “accept or lean towards Platonism,” while 37.7% said they “accept or lean towards nominalism.” The rest (23%) described their position as “other.” However, given that this was a general survey of philosophers, this may overstate the case for “other.” Mark Balaguer reports that “among philosophers of math, I would be stunned if the number were anywhere near that high. Just about everyone in that field is either a Platonist or a nominalist” (personal communication).

^2^A discussion of both positions and their historical and contemporary proponents is Balaguer (2009).

## References

[ref1] Andres M. Ostry D. J. Nicol F. Paus T. (2008). Time course of number magnitude interference during grasping. Cortex 44, 414–419. 10.1016/j.cortex.2007.08.007, PMID: 18387573

[ref2] Balaguer M. (2009). “Realism and anti-realism in mathematics” in Philosophy of mathematics. ed. Irvine A. (Amsterdam: Elsevier), 35–101.

[ref3] Balaguer M. (2014). A guide for the perplexed: what mathematicians need to know to understand philosophers of mathematics. Math. Intell. 36, 3–8. 10.1007/s00283-013-9406-4

[ref4] Balaguer M. (2018). “Fictionalism in the philosophy of mathematics” in The Stanford Encyclopedia of Philosophy (Fall 2018). ed. Zalta E. N. (Palo Alto, CA: Metaphysics Research Lab, Stanford University).

[ref5] Barner D. Alvarez G. Sullivan J. Brooks N. Srinivasan M. Frank M. C. (2016). Learning mathematics in a visuospatial format: a randomized, controlled trial of mental abacus instruction. Child Dev. 87, 1146–1158. 10.1111/cdev.12515, PMID: 27062391

[ref6] Barsalou L. W. (1999). Perceptual symbol systems. Behav. Brain Sci. 22, 577–660. 10.1017/s0140525x99002149, PMID: 11301525

[ref7] Benacerraf P. (1973). Mathematical truth. J. Philos. 70, 661–679. 10.2307/2025075

[ref8] Bender A. Beller S. (2012). Nature and culture of finger counting: diversity and representational effects of an embodied cognitive tool. Cognition 124, 156–182. 10.1016/j.cognition.2012.05.005, PMID: 22695379

[ref9] Bolognesi M. Steen G. (2018). Editors’ introduction: abstract concepts: structure, processing, and modeling. Top. Cogn. Sci. 10, 490–500. 10.1111/tops.12354, PMID: 29932299

[ref10] Borghi A. M. Binkofski F. Castelfranchi C. Cimatti F. Scorolli C. Tummolini L. (2017). The challenge of abstract concepts. Psychol. Bull. 143, 263–292. 10.1037/bul0000089, PMID: 28095000

[ref11] Boroditsky L. Ramscar M. (2002). The roles of body and mind in abstract thought. Psychol. Sci. 13, 185–189. 10.1111/1467-9280.00434, PMID: 11934006

[ref114] Bourget D. Chalmers D. J. (2014). What do philosophers believe? Philos. Stud. 170, 465–500. 10.1007/s11098-013-0259-7

[ref12] Bowin J. (2007). “Aristotelian infinity in Oxford Studies” in Ancient philosophy. ed. Sedley D. (Oxford, UK: Oxford University Press), 233–250.

[ref13] Bueno O. (2013). “Nominalism in the philosophy of mathematics” in The Stanford encyclopedia of philosophy. ed. Zalta E. N. (Palo Alto, CA: Metaphysics Research Lab, Stanford University).

[ref14] Casasanto D. Boroditsky L. (2008). Time in the mind: using space to think about time. Cognition 106, 579–593. 10.1016/j.cognition.2007.03.004, PMID: 17509553

[ref15] Casasanto D. de Bruin A. (2019). Metaphors we learn by: directed motor action improves word learning. Cognition 182, 177–183. 10.1016/j.cognition.2018.09.015, PMID: 30296659

[ref16] Cipora K. Hohol M. Nuerk H. -C. Willmes K. Brożek B. Kucharzyk B.. (2016). Professional mathematicians differ from controls in their spatial-numerical associations. Psychol. Res. 80, 710–726. 10.1007/s00426-015-0677-6, PMID: 26063316 PMC4889706

[ref17] Cipora K. Nuerk H. -C. (2013). Is the SNARC effect related to the level of mathematics? No systematic relationship observed despite more power, more repetitions, and more direct assessment of arithmetic skill. Q. J. Exp. Psychol. 66, 1974–1991. 10.1080/17470218.2013.772215, PMID: 23473520

[ref18] Cipora K. Patro K. Nuerk H. -C. (2015). Are spatial-numerical associations a cornerstone for arithmetic learning? The lack of genuine correlations suggests no. Mind Brain Educ. 9, 190–206. 10.1111/mbe.12093

[ref19] Cipora K. Schroeder P. A. Soltanlou M. Nuerk H. -C. (2018). “More space, better mathematics: is space a powerful tool or a cornerstone for understanding arithmetic?” in Visualizing mathematics. eds. Mix K. S. Battista M. T. (Berlin: Springer), 77–116.

[ref20] Colyvan M. (2012). An introduction to the philosophy of mathematics. Cambridge, UK: Cambridge University Press.

[ref21] Colyvan M. Zalta E. N. (1999). Mathematics: truth and fiction? Review of Mark Balaguer platonism and anti-platonism in mathematics. Philos. Math. 7, 336–349.

[ref22] Comrie B. (1976). Aspect. *Vol* 2 Cambridge, UK: Cambridge University Press.

[ref23] Comrie B. (2013). “Numeral bases” in The world atlas of language structures. eds. Dryer M. S. Haspelmath M. (Leipzig: Max Planck Institute for Evolutionary Anthropology).

[ref24] Cooperrider K. Gentner D. (2019). The career of measurement. Cognition 191:103942. 10.1016/j.cognition.2019.04.011 31302322

[ref25] Costa A. J. Silva J. B. L. Pinheiro-Chagas P. Krinzinger H. Lonnemann J. Willmes K.. (2011). A hand full of numbers: a role for offloading in arithmetics learning? Front. Psychol. 2:368. 10.3389/fpsyg.2011.00368, PMID: 22180748 PMC3235774

[ref26] Dehaene S. Bossini S. Giraux P. (1993). The mental representation of parity and number magnitude. J. Exp. Psychol. Gen. 122, 371–396. 10.1037//0096-3445.122.3.371

[ref27] Domahs F. Krinzinger H. Willmes K. (2008). Mind the gap between both hands: evidence for internal finger-based number representations in children’s mental calculation. Cortex 44, 359–367. 10.1016/j.cortex.2007.08.001, PMID: 18387566

[ref28] Evans V. Green M. (2006). Cognitive linguistics: An introduction. Edinburgh, UK: Edinburgh University Press.

[ref29] Fabry R. E. Pantsar M. (2019). A fresh look at research strategies in computational cognitive science: the case of enculturated mathematical problem solving. Synthese 1–43. 10.1007/s11229-019-02276-9

[ref30] Fauconnier G. Turner M. (1998). Conceptual integration networks. Cogn. Sci. 22, 133–187. 10.1016/S0364-0213(99)80038-X

[ref31] Fausey C. M. Matlock T. (2011). Can grammar win elections? Polit. Psychol. 32, 563–574. 10.1111/j.1467-9221.2010.00802.x

[ref32] Fay A. J. Maner J. K. (2012). Warmth, spatial proximity, and social attachment: the embodied perception of a social metaphor. J. Exp. Soc. Psychol. 48, 1369–1372. 10.1016/j.jesp.2012.05.017

[ref33] Fillmore C. J. (1982). “Frame semantics” in linguistics in the morning calm. Seoul: Hanshin Publishing.

[ref34] Fischer M. H. Brugger P. (2011). When digits help digits: spatial–numerical associations point to finger counting as prime example of embodied cognition. Front. Psychol. 2:260. 10.3389/fpsyg.2011.00260, PMID: 22028696 PMC3198540

[ref35] Fischer M. H. Rottmann J. (2005). Do negative numbers have a place on the mental number line. Psychol. Sci. 47, 22–32.

[ref36] Frank M. C. Barner D. (2012). Representing exact number visually using mental abacus. J. Exp. Psychol. Gen. 141:134. 10.1037/a0024427, PMID: 21767040

[ref37] Frege G. (1884). The basic laws of arithmetic. Berkeley, CA: University of California Press.

[ref38] Gallese V. Lakoff G. (2005). The brain’s concepts: the role of the sensory-motor system in conceptual knowledge. Cogn. Neuropsychol. 22, 455–479. 10.1080/02643290442000310, PMID: 21038261

[ref39] Gibbs R. W. (1994). The poetics of mind: Figurative thought, language, and understanding. Cambridge, UK: Cambridge University Press.

[ref40] Gibbs R. W. (2005). Embodiment and cognitive science. Cambridge, UK: Cambridge University Press.

[ref41] Gibbs R. W. Berg E. A. (1999). Embodied metaphor in perceptual symbols. Behav. Brain Sci. 22, 617–618. 10.1017/S0140525X99312140

[ref42] Gibbs R. W. Lima P. L. C. Francozo E. (2004). Metaphor is grounded in embodied experience. J. Pragmat. 36, 1189–1210. 10.1016/j.pragma.2003.10.009

[ref43] Göbel S. M. Shaki S. Fischer M. H. (2011). The cultural number line: a review of cultural and linguistic influences on the development of number processing. J. Cross Cult. Psychol. 42, 543–565. 10.1177/0022022111406251

[ref44] Goldin-Meadow S. (2005). Hearing gesture: How our hands help us think. Cambridge, MA: Harvard University Press.

[ref45] Grady J. E. (1997). Theories are buildings revisited. Cogn. Linguist. 8, 267–290. 10.1515/cogl.1997.8.4.267

[ref46] Haser V. (2005). Metaphor, metonymy, and experientialist philosophy: Challenging cognitive semantics. Berlin: Walter de Gruyter.

[ref47] Henik A. Tzelgov J. (1982). Is three greater than five: the relation between physical and semantic size in comparison tasks. Mem. Cogn. 10, 389–395. 10.3758/bf03202431, PMID: 7132716

[ref48] Hubbard E. M. Piazza M. Pinel P. Dehaene S. (2005). Interactions between number and space in parietal cortex. Nat. Rev. Neurosci. 6, 435–438. 10.1038/nrn1684, PMID: 15928716

[ref49] Hurewitz F. Gelman R. Schnitzer B. (2006). Sometimes area counts more than number. Proc. Natl. Acad. Sci. U. S. A. 103, 19599–19604. 10.1073/pnas.0609485103, PMID: 17159143 PMC1748271

[ref50] Husserl E. (2012). Logical investigations. *Vol* 1 London, UK: Routledge.

[ref51] Ifrah G. (1998). The universal history of numbers: From pre-history to the invention of the computer. London, UK: Collins and Harvill Press.

[ref52] IJzerman H. Semin G. R. (2010). Temperature perceptions as a ground for social proximity. J. Exp. Soc. Psychol. 46, 867–873. 10.1016/j.jesp.2010.07.015

[ref53] Johnson M. (1987). The body in the mind: The bodily basis of meaning, imagination, and reason. Chicago, IL: University of Chicago Press.

[ref54] Johnson M. Lakoff G. (2002). Why cognitive linguistics requires embodied realism. Cogn. Linguist. 13, 245–264. 10.1515/cogl.2002.016

[ref55] Kertész A. Rákosi C. (2009). Cyclic vs. circular argumentation in the conceptual metaphor theory. Cogn. Linguist. 20, 703–732. 10.1515/COGL.2009.030

[ref56] Knops A. Viarouge A. Dehaene S. (2009). Dynamic representations underlying symbolic and nonsymbolic calculation: evidence from the operational momentum effect. Atten. Percept. Psychophysiol. 71, 803–821. 10.3758/APP.71.4.803, PMID: 19429960

[ref57] Kousta S. -T. Vigliocco G. Vinson D. P. Andrews M. Del Campo E. (2011). The representation of abstract words: why emotion matters. J. Exp. Psychol. Gen. 140, 14–34. 10.1037/a0021446, PMID: 21171803

[ref58] Kövecses Z. (2002). Metaphor: A practical introduction. Oxford, UK: Oxford University Press.

[ref59] Kramer P. Bressan P. Grassi M. (2018). The SNARC effect is associated with worse mathematical intelligence and poorer time estimation. R. Soc. Open Sci. 5:172362. 10.1098/rsos.172362, PMID: 30224999 PMC6124133

[ref60] Kusch M. (2020). “Psychologism” in The Stanford Encyclopedia of Philosophy (Spring 2020). ed. Zalta E. N. (Palo Alto, CA: Metaphysics Research Lab, Stanford University).

[ref61] Lakoff G. (2008). “The neural theory of metaphor” in Cambridge handbook of metaphor and thought. ed. Gibbs R. W. (Cambridge, UK: Cambridge University Press), 17–38.

[ref62] Lakoff G. (2012). Explaining embodied cognition results. Top. Cogn. Sci. 4, 773–785. 10.1111/j.1756-8765.2012.01222.x, PMID: 22961950

[ref63] Lakoff G. Johnson M. (1980). Metaphors we live by. Chicago, IL: University of Chicago Press.

[ref64] Lakoff G. Johnson M. (1999). Philosophy in the flesh: The embodied mind and its challenge to western thought. New York: Basic Books.

[ref65] Lakoff G. Núñez R. E. (2000). Where mathematics comes from: How the embodied mind brings mathematics into being. New York: Basic Books.

[ref66] Landau M. J. (2017). Conceptual metaphor in social psychology: The poetics of everyday life. London: Routledge.

[ref67] Landau M. J. Meier B. P. Keefer L. A. (2010). A metaphor-enriched social cognition. Psychol. Bull. 136, 1045–1067. 10.1037/a0020970, PMID: 20822208

[ref68] Linnebo Ø. (2018). “Platonism in the philosophy of mathematics” in The Stanford Encyclopedia of Philosophy (Spring 2018). ed. Zalta E. N. (Palo Alto, CA: Metaphysics Research Lab, Stanford University).

[ref69] Lupyan G. Winter B. (2018). Language is more abstract than you think, or, why aren’t languages more iconic? Philos. Trans. R. Soc. Lond. Ser. B Biol. Sci. 373:20170137. 10.1098/rstb.2017.0137, PMID: 29915005 PMC6015821

[ref70] Marghetis T. (2015). Every number in its place: the spatial foundations of calculation and conceptualization. [PhD Thesis]. UC San Diego.

[ref71] Marghetis T. Eberle L. Bergen B. (2015). “The mental number-line spreads by gestural contagion.” in *Proceedings of the 37th Annual Meeting of the Cognitive Science Society*; July 22–25, 2015; Pasadena, California.

[ref72] Marghetis T. Núñez R. (2013). The motion behind the symbols: a vital role for dynamism in the conceptualization of limits and continuity in expert mathematics. Top. Cogn. Sci. 5, 299–316. 10.1111/tops.12013, PMID: 23460466

[ref73] Marghetis T. Núñez R. Bergen B. K. (2014). Doing arithmetic by hand: hand movements during exact arithmetic reveal systematic, dynamic spatial processing. Q. J. Exp. Psychol. 67, 1579–1596. 10.1080/17470218.2014.897359, PMID: 25051274

[ref74] Marghetis T. Youngstrom K. (2014). “Pierced by the number line: Integers are associated with back-to-front sagittal space.” in *Proceedings of the 36th Annual Meeting of the Cognitive Science Society*; July 23-26, 2014; Quebec City, Canada, 36.

[ref75] Matlock T. (2004). Fictive motion as cognitive simulation. Mem. Cogn. 32, 1389–1400. 10.3758/bf03206329, PMID: 15900932

[ref76] Matlock T. (2011). “The conceptual motivation of aspect” in Motivation in grammar and the lexicon. eds. Panther K. -U. Radden G. (Amsterdam: John Benjamins), 133–147.

[ref77] McCrink K. Dehaene S. Dehaene-Lambertz G. (2007). Moving along the number line: operational momentum in nonsymbolic arithmetic. Percept. Psychophys. 69, 1324–1333. 10.3758/bf03192949, PMID: 18078224

[ref78] McNeill D. (1992). Hand and mind: What gestures reveal about thought. Chicago, IL: University of Chicago Press.

[ref79] Murphy G. L. (1996). On metaphoric representation. Cognition 60, 173–204. 10.1016/0010-0277(96)00711-1 8811744

[ref80] Murphy G. L. (1997). Reasons to doubt the present evidence for metaphoric representation. Cognition 62, 99–108. 10.1016/s0010-0277(96)00725-1, PMID: 8997172

[ref81] Noël M. -P. (2005). Finger gnosia: a predictor of numerical abilities in children? Child Neuropsychol. 11, 413–430. 10.1080/09297040590951550, PMID: 16306017

[ref82] Núñez R. E. (2005). Creating mathematical infinities: metaphor, blending, and the beauty of transfinite cardinals. J. Pragmat. 37, 1717–1741. 10.1016/j.pragma.2004.09.013

[ref83] Núñez R. (2008a). “A fresh look at the foundations of mathematics: gesture and the psychological reality of conceptual metaphor” in Metaphor and gesture. eds. Cienki A. Müller C. (Amsterdam: John Benjamins), 93–114.

[ref84] Núñez R. (2008b). “Conceptual metaphor, human cognition, and the nature of mathematics” in The Cambridge handbook of metaphor and thought. ed. Gibbs R. W. (Cambridge, UK: Cambridge University Press), 339–362.

[ref85] Pantsar M. (2015). In search of ℵ_0_: how infinity can be created. Synthese 192, 2489–2511. 10.1007/s11229-015-0775-4

[ref86] Pantsar M. (2018). Early numerical cognition and mathematical processes. Theoria 33, 285–304. 10.1387/theoria.17682

[ref87] Pecher D. Boot I. (2011). Numbers in space: differences between concrete and abstract situations. Front. Psychol. 2:121. 10.3389/fpsyg.2011.00121, PMID: 21713061 PMC3114072

[ref88] Pelletier F. J. Elio R. Hanson P. (2008). Is logic all in our heads? From naturalism to psychologism. Stud. Logica. 88, 3–66. 10.1007/s11225-008-9098-5

[ref89] Putnam H. (1975). What is mathematical truth? Hist. Math. 2, 529–533. 10.1016/0315-0860(75)90116-0

[ref90] Quine W. V. O. (1980). From a logical point of view: 9 logico-philosophical essays. Cambridge, MA: Harvard University Press.

[ref91] Rakova M. (2002). The philosophy of embodied realism: a high price to pay? Cogn. Linguist. 13, 215–244. 10.1515/cogl.2002.015

[ref92] Reeve R. (2011). Five-to 7-year-olds’ finger gnosia and calculation abilities. Front. Psychol. 2:359. 10.3389/fpsyg.2011.00359, PMID: 22171220 PMC3236444

[ref93] Sell A. J. Kaschak M. P. (2012). The comprehension of sentences involving quantity information affects responses on the up-down axis. Psychon. Bull. Rev. 19, 708–714. 10.3758/s13423-012-0263-5, PMID: 22588974 PMC5101539

[ref94] Shaki S. Fischer M. H. Petrusic W. M. (2009). Reading habits for both words and numbers contribute to the SNARC effect. Psychon. Bull. Rev. 16, 328–331. 10.3758/PBR.16.2.328, PMID: 19293102

[ref95] Shaki S. Petrusic W. M. (2005). On the mental representation of negative numbers: context-dependent SNARC effects with comparative judgments. Psychon. Bull. Rev. 12, 931–937. 10.3758/bf03196788, PMID: 16524013

[ref96] Shapiro S. (1996). Mathematical structuralism. Philos. Math. 4, 81–82.

[ref97] Sixtus E. Fischer M. H. Lindemann O. (2017). Finger posing primes number comprehension. Cogn. Process. 18, 237–248. 10.1007/s10339-017-0804-y, PMID: 28374126

[ref98] Sixtus E. Lindemann O. Fischer M. H. (2018). Stimulating numbers: signatures of finger counting in numerosity processing. Psychol. Res. 84, 152–167. 10.1007/s00426-018-0982-y, PMID: 29344725

[ref99] Spivey M. (2007). The continuity of mind. Oxford, UK: Oxford University Press.

[ref100] Staats S. Batteen C. (2009). Stretching, sliding and strategy: indexicality in algebraic explanations. Res. Math. Educ. 11, 57–71. 10.1080/14794800902732225

[ref101] Stigler J. W. (1984). “Mental abacus”: the effect of abacus training on Chinese children’s mental calculation. Cogn. Psychol. 16, 145–176. 10.1016/0010-0285(84)90006-9

[ref102] Thibodeau P. H. Boroditsky L. (2011). Metaphors we think with: the role of metaphor in reasoning. PLoS One 6:e16782. 10.1371/journal.pone.0016782, PMID: 21373643 PMC3044156

[ref103] Vigliocco G. Kousta S. -T. Della Rosa P. A. Vinson D. P. Tettamanti M. Devlin J. T.. (2013). The neural representation of abstract words: the role of emotion. Cereb. Cortex 24, 1767–1777. 10.1093/cercor/bht025, PMID: 23408565

[ref104] Weir A. (2020). “Formalism in the philosophy of mathematics” in The Stanford Encyclopedia of Philosophy (Spring 2020). ed. Zalta E. N. (Palo Alto, CA: Metaphysics Research Lab, Stanford University).

[ref105] Wilson M. (2002). Six views of embodied cognition. Psychon. Bull. Rev. 9, 625–636. 10.3758/bf03196322, PMID: 12613670

[ref106] Wilson R. A. Foglia L. (2015). “Embodied cognition” in The Stanford Encyclopedia of Philosophy. ed. Zalta E. N. (Palo Alto, CA: Metaphysics Research Lab).

[ref107] Wilson A. D. Golonka S. (2013). Embodied cognition is not what you think it is. Front. Psychol. 4:58. 10.3389/fpsyg.2013.00058, PMID: 23408669 PMC3569617

[ref108] Winter B. Marghetis T. Matlock T. (2015). Of magnitudes and metaphors: explaining cognitive interactions between space, time, and number. Cortex 64, 209–224. 10.1016/j.cortex.2014.10.015, PMID: 25437376

[ref109] Winter B. Perlman M. Matlock T. (2013). Using space to talk and gesture about numbers: evidence from the TV news archive. Gesture 13, 377–408. 10.1075/gest.13.3.06win

[ref110] Wood G. Willmes K. Nuerk H. -C. Fischer M. H. (2008). On the cognitive link between space and number: a meta-analysis of the SNARC effect. Psychol. Sci. 50, 489–525.

[ref111] Woodin G. Winter B. (2018). Placing abstract concepts in space: quantity, time and emotional valence. Front. Psychol. 9:2169. 10.3389/fpsyg.2018.02169, PMID: 30487766 PMC6246627

[ref112] Yoshimi J. (2012). Active internalism and open dynamical systems. Philos. Psychol. 25, 1–24. 10.1080/09515089.2011.569919

[ref113] Zebian S. (2005). Linkages between number concepts, spatial thinking, and directionality of writing: the SNARC effect and the reverse SNARC effect in English and Arabic monoliterates, biliterates, and illiterate Arabic speakers. J. Cogn. Cult. 5, 165–190. 10.1163/1568537054068660

